# Cell metabolomics to study the function mechanism of *Cyperus rotundus* L. on triple-negative breast cancer cells

**DOI:** 10.1186/s12906-020-02981-w

**Published:** 2020-08-26

**Authors:** Shuangshuang Ma, Fukai Wang, Caijuan Zhang, Xinzhao Wang, Xueyong Wang, Zhiyong Yu

**Affiliations:** 1 Shandong Cancer Hospital and Institute, Shandong First Medical University and Shandong Academy of Medical Sciences, No.440 jiyan road, Jinan, 250017 Shandong China; 2 Shandong Hongjitang Pharmaceutical Group Co.,Ltd., Jinan, 250000 China; 3 School of life Science, Beijing University of Chinese Medicine, Northeast corner of intersection of Sunshine South Street and Baiyang East Road, Fang-Shan District, Beijing, 102488 China; 4 School of Chinese Materia Medical, Beijing University of Chinese Medicine, No.11 North 3rd Ring East Road, Chao-Yang District, Beijing, 100029 China

**Keywords:** Cell metabolomics, Triple-negative breast cancer, *Cyperus rotundus* L., Aerobic glycolysis, UPLC-Q-TOF-MS/MS

## Abstract

**Background:**

Triple-negative breast cancer (TNBC) is a kind of malignant tumor with higher recurrence and metastasis rate. According to historical records, the dry rhizomes *Cyperus rotundus* L. could be ground into powder and mixed with ginger juice and wine for external application for breast cancer. We studied the effect of the ethanol extract of *Cyperus rotundus* L. (EECR) on TNBC cells and found its’ apoptosis-inducing effect with a dose-relationship. But the function mechanism of EECR on TNBC is still mysterious. Hence, the present research aimed to detect its function mechanism at the small molecule level through ultra-high performance liquid chromatography coupled with quadrupole-time-of-flight mass spectrometry (UPLC-Q-TOF-MS/MS) metabolomics.

**Methods:**

The CCK-8 assay and the Annexin V-FITC/PI assay were applied to test the effect of EECR on MDA-MB-231 cells and MDA-MB 468 cells at various concentrations of 0, 200, 400, and 600 μg/ml. UPLC-Q-TOF-MS/MS based metabolomics was used between the control group and the EECR treatment groups. Multivariate statistical analysis was used to visualize the apoptosis-inducing action of EECR and filtrate significantly changed metabolites.

**Results:**

The apoptosis-inducing action was confirmed and forty-nine significantly changed metabolites (VIP > 1, *p* < 0.05, and FC > 1.2 or FC < 0.8) were identified after the interference of EECR. The level of significant differential metabolites between control group, middle dose group, and high dose group were compared and found that which supported the apoptosis-inducing action with dose-dependence.

**Conclusion:**

By means of metabolism, we have detected the mechanism of EECR inducing apoptosis of TNBC cells at the level of small molecule metabolites and found that EECR impacted the energy metabolism of TNBC cells. In addition, we concluded that EECR induced apoptosis by breaking the balance between ATP-production and ATP-consumption: arresting the pathways of Carbohydrate metabolism such as Central carbon metabolism in cancer, aerobic glycolysis, and Amino sugar and nucleotide sugar metabolism, whereas accelerating the pathways of ATP-consumption including Amino Acids metabolism, Fatty acid metabolism, Riboflavin metabolism and Purine metabolism. Although further study is still needed, EECR has great potential in the clinical treatment of TNBC with fewer toxic and side effects.

## Background

According to WHO reports, breast cancer involving 2.09 million cases is the most common cancer in 2018 and seriously affects woman’s health around the world. Although breast cancer has a high cure rate when detected early and treated according to best practices, triple-negative breast cancer (TNBC) is an exception due to its lack expression characteristic of estrogen receptor (ER), progesterone receptor (PR), and human epidermal growth factor receptor-2 (Her-2). Patients with TNBC have an increased likelihood of distant recurrence and death. In addition, they cannot be treated with endocrine therapy or Her-2 targeted therapies [1, 2]. The severe side-effect of chemotherapy drugs is almost universal and obstructs the treatment of cancer. Along with an earlier age-of-onset and more probability of the ‘triple-negative’ phenotype, an effective drug with low toxicity is always in demand during the treatment of breast cancer [3–5].

The dry rhizomes of *Cyperus rotundus* L., also named Xiangfu in Chinese, have an application history of 1700 years in China and are mainly applied to treat gynecological diseases. According to ancient literature, Xiangfu could be ground into powder and mixed with ginger juice and wine for external application for breast cancer. Presently, the cytotoxic effect of *Cyperus rotundus* L. on breast cancer cell lines has been reported [6–9]. In our previous study, we identified 21 phytochemical compounds in the ethanol extract *Cyperus rotundus* L.(EECR) and found that EECR induced TNBC cell apoptosis which is reflected by the enhancement of ratio of the Bax/Bcl-2 [10]. Despite the distinct apoptosis-inducing action, the function mechanism of EECR on TNBC cells is still mysterious and needs further in-deep research.

Metabolites being free from the impacts of epigenetic regulation and post-translational modifications act as the direct form for their biochemical activity, and hence they more veritably reflect their phenotype [11]. UPLC-MS/MS has the advantages of simple pretreatment, high sensitivity, and the remarkable capacity of detection and identification, so it can be applied for complex samples to collect as much as features [12]. Accompanied with the development of high-resolution mass spectrometer, it shows a major advantage for untargeted metabolomics and is widely applied to detect the specific metabolic changes of a biological system in responding to disease, infection, drugs, or toxins [13–18].

In this research, UPLC-Q-TOF-MS/MS based cell metabolomics combined with a serious of metabolomics software such as Human Metabolome Database (http://www.hmdb.ca/), KEGG Database (https://www.kegg.jp/), MetaboAnalyst (https://www.metaboanalyst.ca/), and XCMS Online (https://xcmsonline.scripps.edu/), was used for the detection and identification of features of cell extraction. The multivariate statistical discriminate analysis was applied for searching significant differential metabolites of TNBC cell lines after the intervention of EECR. The student’s T-test was adopted for explaining the changes of each metabolite at the status of low, medium and high concentrations. TNBC cell lines of MDA-MB-468 and MDA-MB-231 were used at the same treatment condition aiming to increase the credibility of the results.

Based on the above analysis and our previously researches, the main objective of this study was to detect the significantly changes of metabolites after the intervention of EECR, reveal the function mechanism of EECR on inducing apoptosis of TNBC cells at the level of small molecule metabolites, and explain the nature of its dose-dependent apoptosis-inducing character. And then, it could lay the research foundation for the further deep study of EECR and provide a guide for clinical application.

## Methods

### Materials and chemicals

The dry rhizomes of *Cyperus rotundus* L. were purchased from Anhui Xiehecheng Co., Ltd. (Bozhou, China); the ethanol extract of *Cyperus rotundus* L. was acquired referring to previous reporting method [10]; Dulbecco’s modified Eagle’s medium (DMEM), penicillin, and streptomycin were purchased from Nanjing KeyGen Biotech. Co. Ltd. (Jiangsu, China); Fetal bovine serum (FBS) and Phosphate-buffered saline (PBS) was purchased from Gibco Company (New York, U.S.A.); Dimethyl sulfoxide (DMSO) was purchased from Thermo Fisher Scientific (Massachusetts, U.S.A.); The Cell Counting Kit-8 assay (CCK-8) was purchased from MedChemExpress (New Jersey, U.S.A.); Acetonitrile (liquid chromatogram grade) was purchased from Merck KGaA (Darmstadt, Germany); Formic acid was purchased from Honeywell Trading Co., Ltd. (Muskegon, USA).

### Mass spectrometry analysis of EECR

The EECR had been analyzed by UPLC-Q-TOF MS/MS for quality control and 21 metabolites were listed in previous study. The detail information was shown in the article [10].

### Cell treatment and viability analysis

Cell lines of MDA-MB-468 and MDA-MB-231 were obtained from the Cell Bank of Shanghai Institute of Cell Biology (Chinese Academy of Sciences) and cultured in the air with 5% CO_2_ at 37 °C. The cells were grown in DMEM supplemented with 10% (v/v) FBS and 100 units/mL penicillin and streptomycin. 1 × 10^4^ cells per well were seeded in 96-well plates and cultured for 24 h. Subsequently, MDA-MB-468 cells and MDA-MB-231 cells were respectively treated with EECR at various concentrations of 0, 200, 400, and 600 μg/ml. All cells were exposed to EECR with mentioned concentrations for another 24 h. The cell viability was conducted according to the manufacturer’s instruction of CCK-8 assay. The optical density (OD) was measured at 450 nm.

### Flow cytometry

For confirming the apoptosis induced by EECR, three concentrations of EECR (0, 200, and 400 μg/ml) were chosen to treat MDA-MB-231 cell, however, three concentrations (0, 400, and 600 μg/ml) were selected for MDA-MB-468. Cells at the density of 1 × 10^4^ cells/well were seeded in 6-well plates and cultured. Until grew over 75% of the bottom, the cells were exposed to EECR at mentioned above concentrations for 24 h. The cells were collected by EDTA-free trypsin, centrifuged at 1000 rpm for 3 min, and washed twice with cold PBS softly. Following the instructions, the cells were re-suspended at a density of 1× 10^6^ cells/ml. 100 μl suspension was mixed with 5 μl of Annexin V-FITC and 5 μl of Propidium Iodide (PI), incubated at 4 °C for 30 min in the dark, and tested on the FACS Calibur (Becton Dickinson, USA).

### Cell pretreatment and metabolomics analysis

MDA-MB-468 and MDA-MB-231 cells were pooled onto culture dishes (5 cm in diameter) at a density of 1 × 10^7^ cells per dish. After cultured for 24 h, the culture mediums were replaced for new blank culture medium (Control group) or medium containing EECR at various concentrations (Treatment group), and training for another 24 h. Three repeats were set each group. All cells were washed thrice with ice-cold PBS and quenched by liquid nitrogen. Using ice-cold methanol/water (80:20; v/v) as the extraction solution, the cells were scraped with cell Scraper, transferred to pre-cooling centrifuge tubes, intermittently vortex for 3 min, and centrifuged (14,000 rpm, 4 °C) for 5 min. The supernatants were filtered with 0.22 μm microfiltration membranes and moved to new tubes for metabolomics analysis. The quality control (QC) samples were earned by equally mixing each sample. At first, six QC samples were operated to detect the stability of the instrument, and one QC sample was run after every ten injections during all sequences.

The supernatants were separated on the C18 column (2.1 × 100 mm, 1.8 μm, Acquity UPLC HSS T3, USA) at 40 °C and a flow rate of 0.3 mL/min by the UPLC system (Waters Acquity UPLC class, U.S.A.). The temperature of the sample plate was set at 8 °C and the injection volume was 5 μL. The mobile phase A was water with 0.1% formic acid and the mobile phase B was acetonitrile. The gradient elution was adopted as follows: 0–1 min, 5% B; 1–2 min, 5–25% B; 2–7 min, 24–40% B; 7–8.5 min, 40–95% B; 8.5–13.5 min, 95% B; 13.5–14 min, 95–5% B; 14–18 min, 5% B. The mass spectrometry information was detected by Q-TOF mass spectrometer equipped with ESI interface (Bruker Impact II™, Germany). Both positive and negative ion modes were used. Capillary voltages were 3500 V (in positive ion mode) and 3000 V (in negative ion mode), nebulizer pressure was 2.0 Bar, the flow of dry gas was 8.0 l/min, and the temperature of dry gas was 200 °C. The mass range of full-scan was set from 50 to 1000 Da. Na formate correction fluid was used for ensuring accurate mass data.

### Data analysis

After the calibration, the raw data was converted into mzXML data by Burker Compass DataAnalysis (Germany). Subsequently, the peak information of mass, retention time, and intensity (> 10^4^) were exported in the form of .xls data through XCMS Online (https://xcmsonline.scripps.edu/). Multivariate statistical discriminate analysis, such as principal component analysis (PCA) and partial least squares discriminant analysis (PLS-DA), were carried out on SIMCA-P (version 13.0, Umetrics, Sweden). The PCA, an unsupervised pattern recognition method, was applied to observe the distribution of the initial data. The cross-validated PLS-DA was used to screen the different metabolites between treatment and control groups (VIP > 1), and the permutations plot was used for assessing the PLS-DA model. Fold Change (FC) expresses the ratio of peak area of metabolites in the treatment and control group, which is used for filtrating metabolites with more significant difference. Student’s t-test and FC were employed for defining significantly difference (*p* < 0.05, FC < 0.8 or FC > 1.2).

## Result

### The cytotoxicity analysis of EECR on TNBC cells

**Fig. 1:**
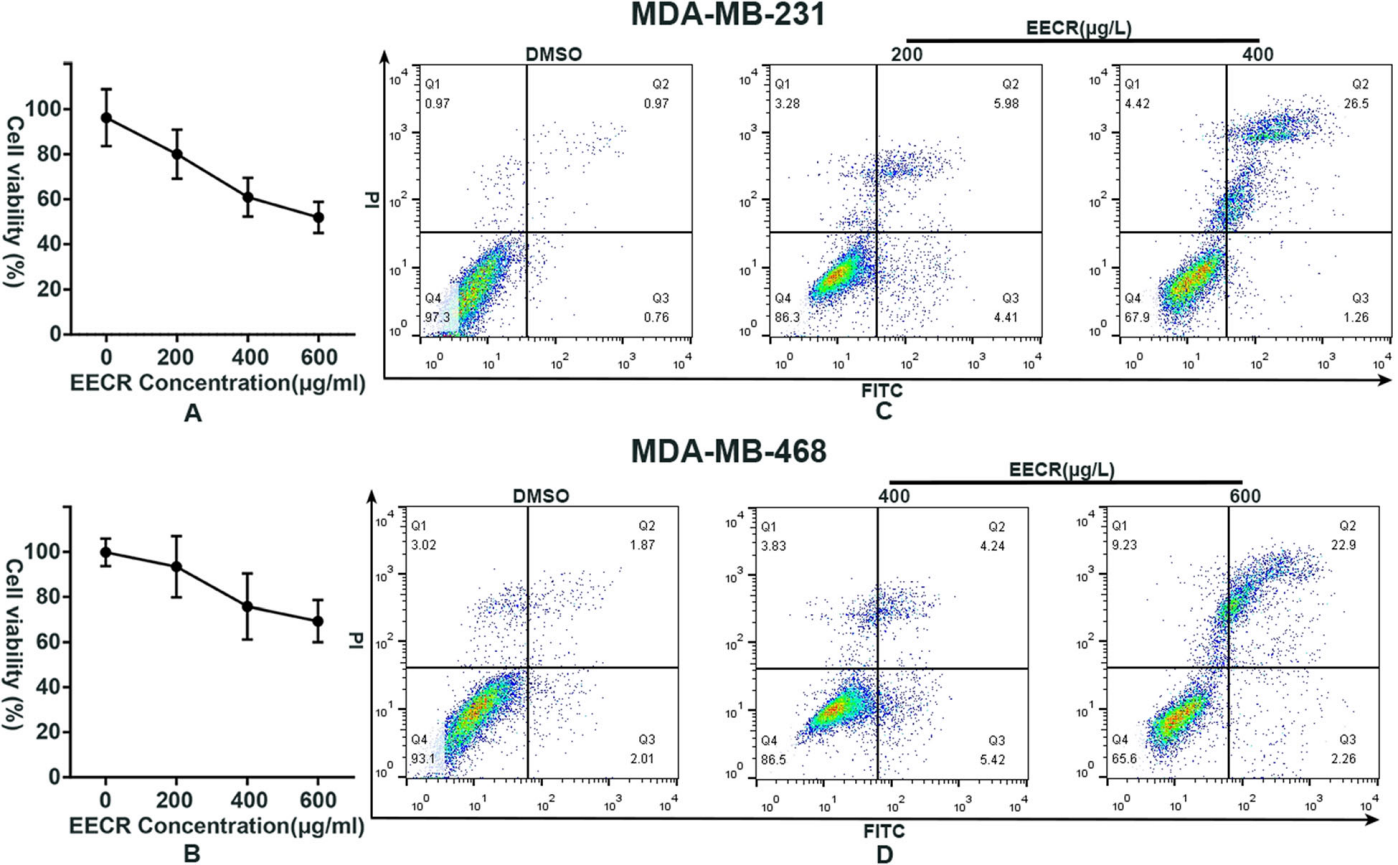
EECR induces apoptosis of TNBC cells with dose-dependent activity. CCK8 assay was used to test the cell viability of TNBC cells (**a**: MDA-MB-231cell line, **b**: MDA-MB-468 cell line). Contrasting with the control group, the cell viability of every group was exhibited in a percentage form and decreased linearly along with the addition of EECR concentrations (**a**, **b**). The Annexin V-FITC/PI assay was employed in attending to the test of apoptosis. Q1 quadrant represents necrotic cells, Q2 represents late apoptotic cells, Q3 represents early apoptotic cells, and Q4 represents normal cells. In MDA-MB-231 cell line, the sum of Q2 and Q3 was 1.73% in the control group, 10.39% in the 231–200 group, and 23.76% in the 231–400 group. In MDA-MB-468 cell line, the sum of Q2 and Q3 was 3.88% in the control group, 9.66% in the 468–400 group, and 25.16% in the 468–600 group

The EECR remarkably inhibits the proliferation of MDA-MB-231 and MDA-MB-468 cells at every concentration mentioned. After the treatment of EECR, the cell viability was down-regulation and remarkably relevant to EECR’s concentrations (Fig. 1 a, b), which is consistent with our previous study. The IC_50_ of EECR on MDA-MB 231 and MDA-MB 468 cell lines were 537.5 μg/ml and 773.3 μg/ml respectively. For maintaining the similar cell viability between these two cell lines during the stage of metabolism, we respectively chose the concentrations of 400 μg/ml and 600 μg/ml for detecting the EECR function mechanism on TNBC cells. In addition to explaining the reasons of dose-related apoptotic effects on TNBC cells, the EECR concentrations of 200 and 400 μg/ml were selected for the following researches in MDA-MB 231 cell line, while the concentrations of 400 and 600 μg/ml were chosen in MDA-MB 468 cell line. It was verified by flow cytometry that the EECR induced apoptosis of TNBC cells in a dose-dependent manner. As shown in Fig. 1, Q1 quadrant represents necrotic cells, Q2 represents late apoptotic cells, Q3 represents early apoptotic cells, and Q4 represents normal cells. The proportion of Q3 in the group of middle-dose of EECR was obviously more than the groups of control and high-dose, which meant that the early apoptotic cells were mainly detected at the middle concentration. While the proportion of Q2 of EECR treatment groups were both bigger than the control groups and kept the consistent relationship with the concentration of EECR which conformed with the result of cell viability analysis.

### Multivariate statistical analysis

**Fig. 2:**
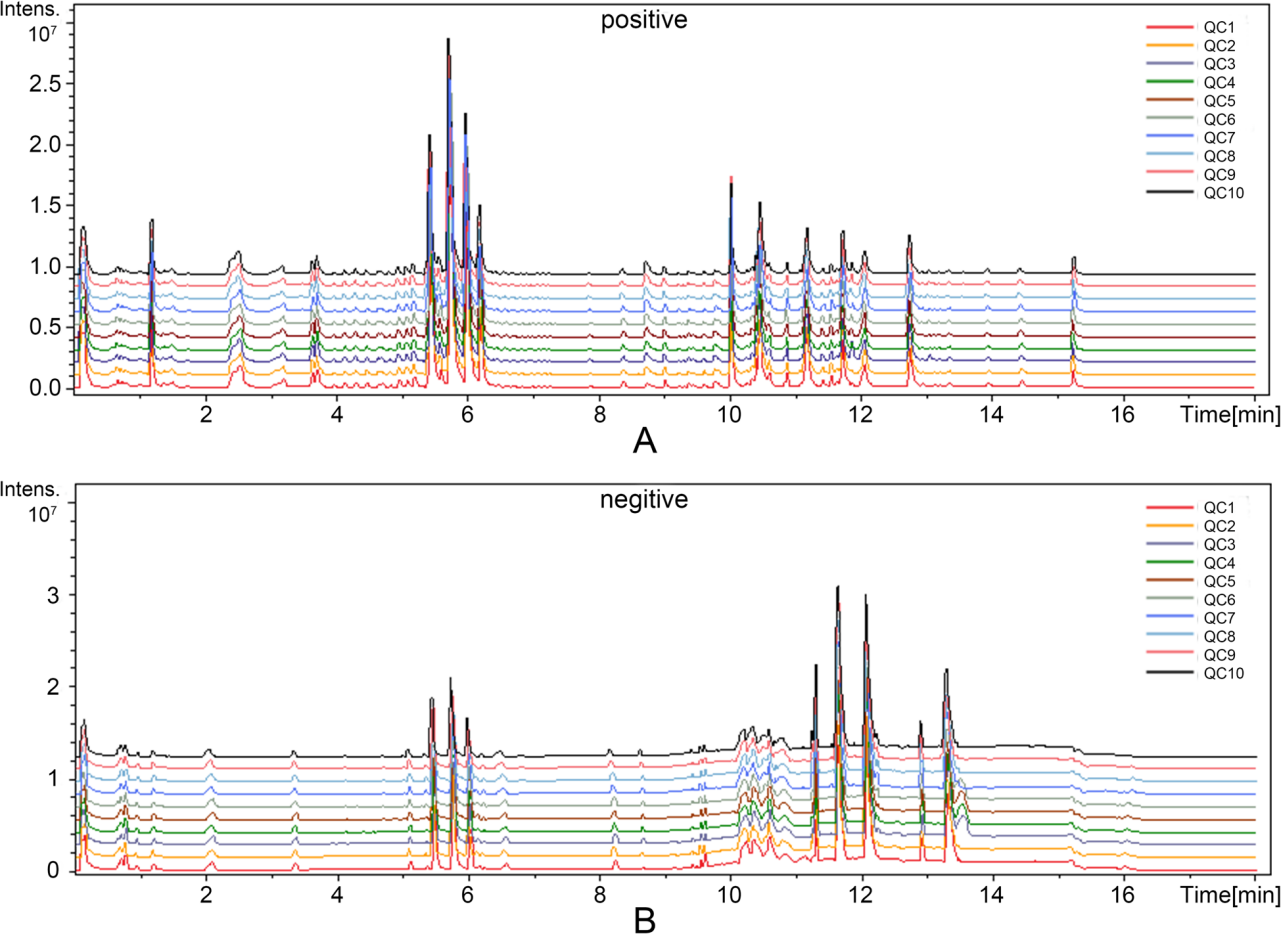
Base peak chromatograms of QCs in ESI^+/−^ mode. **a** shows the BPCs of QCs in positive mode; **b** displays the BPCs of QCs in negative mode. Both two have great repeatability

The base peak chromatograms (BPCs) of QCs were listed in Fig. 2 and the relative standard deviation (RSD) of every metabolite, at the range of 1.95 ~ 22.85% in negative mode and 1.33 ~ 22.30% in positive mode, was listed in Table 1. As listed, the real-time and peak intensity in this experiment both have good repeatability.

**Table 1:** The detailed list of significantly differential metabolites identified in EECR treatment groups in both MDA-MB-231 cell line and MDA-MB-468 cell line

ID	Compound Name	KEGG ID	M/Z	Real time	Adducts	ESI mode	Changes in MDA- MB-231 cells line	Changes in MDA- MB-468 cells line	RSD	Relatd pathway
1	L-Methionine S-oxide	C02989	165.0411	10.61	M(C13)-H[−]	neg	↑*	–	21.85%	Cysteine and methionine metabolism
2	Hexadecanoic acid	C00249	255.2333	12.09	M-H[−]	neg	↑*	–	10.12%	Fatty acid biosynthesis
3	Scopoletin	C01752	232.0595	2.10	M + ACN-H[−]	neg	↑**	–	6.35%	Phenylpropanoid biosynthesis
4	3,4-Dihydroxyphenyllactic acid	C01207	163.0392	8.39	M-H4O2 + H[1+]	pos	↑**	–	17.43%	/
5	O-Butanoylcarnitine	C02862	232.1547	3.47	M + H[1+]	pos	↑**	–	11.86%	/
6	4-Nitrophenol	C00870	138.0198	6.32	M-H[−]	neg	↑**	–	10.59%	Aminobenzoate degradation
7	Vaniline	C00755	151.0402	6.34	M-H[−]	neg	↑**	–	3.45%	Aminobenzoate degradation
8	L-Phenylalanine	C00079	164.0719	2.08	M-H[−]	neg	↑**	–	1.95%	Central carbon metabolism in cancer/ Phenylalanine metabolism
9	Octadecanoic acid	C01530	283.2645	13.31	M-H[−]	neg	↑**	–	7.83%	Fatty acid biosynthesis
10	3-Dehydrosphinganine	C02934	282.2793	11.58	M-H2O + H[1+]	pos	↑**	–	7.74%	Sphingolipid metabolism
11	2-Ethylhexyl phthalate	C03343	279.1593	13.43	M + H[1+],M-H[−]	pos/neg	↑**	↓	11.19%	/
12	L-Homocarnosine	C00884	195.1229	3.23	M-HCOOH+H[1+]	pos	↑**	↓	13.60%	Arginine and proline metabolism
13	Melatonin	C01598	269.0860	9.24	M + Cl37[−]	neg	↑**	↓	7.70%	Tryptophan metabolism
14	beta-D-Galactose	C00962	219.0267	0.78	M + K[1+]	pos	↓**	–	2.15%	/
15	beta-L-Aspartylhydroxamate	C03124	226.9662	1.60	M + Br[−]	neg	↓**	–	19.55%	/
16	Adenine	C00147	136.0620	1.24	M + H[1+]	pos	–	↑**	8.19%	Purine metabolism
17	Citric acid	C00158	191.0199	0.73	M-H[−],M + Na[+]	neg/pos	–	↑**	7.30%	Central carbon metabolism in cancer
18	L-Tyrosine	C00082	182.0813	1.03	M + H[1+]	pos	–	↑**	10.23%	Central carbon metabolism in cancer/Tyrosine metabolism
19	2-Phenylacetamide	C02505	136.0757	0.85	M + H[1+]	pos	–	↑**	8.61%	Phenylalanine metabolism
20	Riboflavin-5-phosphate	C00061	455.0977	4.15	M-H[−]	neg	–	↑**	3.88%	Riboflavin metabolism
21	Flavin adenine dinucleotide	C00016	784.1504	3.81	M-H[−]	neg	–	↑**	6.87%	Riboflavin metabolism
22	4-Hydroxybenzoate	C00156	137.0246	3.90	M-H[−]	neg	–	↓*	4.74%	Benzoate degradation
23	Methyl oleate	C03425	295.2647	12.80	M-H[−]	neg	–	↓**	16.74%	/
24	UDP-N-acetylglucosamine	C00043	606.0748	0.71	M-H[−]	neg	–	↓**	4.06%	Amino sugar and nucleotide sugar metabolism
25	S-Adenosylmethioninamine	C01137	355.1531	12.07	M[1+]	pos	–	↓**	11.94%	Cysteine and methionine metabolism/Arginine and proline metabolism
26	Adenosine	C00212	266.0900	2.90	M-H[−],M + Cl[−],M + H[1+]	neg/pos	–	↓**	8.66%	Purine metabolism
27	(R)-5-Phosphomevalonate	C01107	212.0202	8.39	M-NH3 + H[1+]	pos	↑*	↑**	3.13%	/
28	Hippurate	C01586	178.0512	3.42	M-H[−]	neg	↑**	↑*	12.38%	Phenylalanine metabolism
29	Glutathione	C00051	308.0911	1.24	M + H[1+]	pos	↑**	↑**	1.33%	Cysteine and methionine metabolism
30	Riboflavin	C00255	377.1459	4.19	M + H[1+]	pos	↑**	↑**	3.20%	Riboflavin metabolism
31	Androstanedione	C00674	307.2271	11.33	M + H2O + H[1+]	pos	↑**	↑**	5.41%	Steroid hormone biosynthesis
32	N-Acetyl-L-tyrosine ethyl ester	C01657	208.1336	9.03	M-CO2 + H[1+]	pos	↑**	↑**	6.26%	/
33	Geranylgeranyl diphosphate	C00353	383.2098	10.90	M-HCOONa+H[1+]	pos	↑**	↑**	2.17%	/
34	1-Palmitoylglycerophosphocholine	C04102	496.3401	10.36	M[1+]	pos	↑**	↑**	5.38%	/
35	L-Leucine	C00123	132.1019	0.89	M + H[1+]	pos	↑**	↑**	5.00%	Central carbon metabolism in cancer
36	L-alpha-Aminoadipate	C00956	116.0706	0.82	M-HCOOH+H[1+]	pos	↑**	↑**	1.56%	Lysine degradation
37	1-Methylnicotinamide	C02918	137.0710	0.79	M[1+]	pos	↑**	↑**	1.48%	Nicotinate and nicotinamide metabolism
38	(R)-4′-Phosphopantothenoyl-L-cysteine	C04352	335.1067	7.89	M-HCOONa+H[1+],M-HCOOH+H[1+]	pos	↑**	↑**	4.32%	Pantothenate and CoA biosynthesis
39	Inosine	C00294	249.0632	3.00	M-H2O-H[−]	neg	↑**	↑**	5.46%	Purine metabolism
40	Cytosine	C00380	146.9832	0.82	M + K-2H[−]	neg	↑**	↑**	3.88%	Pyrimidine metabolism
41	Indole-3-acetate	C00954	176.0710	7.89	M + H[1+]	pos	↑**	↑**	3.36%	Tryptophan metabolism
42	Pantothenate	C00864	202.1077	3.22	M-H2O + H[1+],M + H[1+]	pos	↑**	↓**	20.56%	Pantothenate and CoA biosynthesis
43	L-Carnitine	C00318	162.1125	0.80	M-H[−]	pos	↓**	↓**	2.31%	/
44	Choline phosphate	C00588	184.0734	0.79	M+	pos	↓**	↓**	4.06%	/
45	beta-D-Fructose	C02336	203.0527	0.80	M + Na[1+]	pos	↓**	↓**	3.18%	Amino sugar and nucleotide sugar metabolism
46	N-Acetylneuraminate	C00270	292.1031	3.52	M-H2O + H[1+]	pos	↓**	↓**	2.94%	Amino sugar and nucleotide sugar metabolism
47	D-Glucose	C00031	215.0330	0.80	M + Cl[−]	neg	↓**	↓**	4.13%	Central carbon metabolism in cancer
48	L-Glutamate	C00025	146.0460	0.74	M-H[−]	neg	↓**	↓**	2.67%	Central carbon metabolism in cancer
49	N-Succinyl-L,L-2,6-diaminopimelate	C04421	274.0926	3.52	M-NH3 + H[1+]	pos	↓**	↓**	3.37%	Lysine biosynthesis

↑ means that the level of the corresponding metabolite is higher in EECR treatment group compared to the control group; ↓ means that the level of the metabolite is lower in EECR treatment group compared to the control group. - indicates that no distinct change between the EECR treatment groups and the control group is found. / represents that the pathway what the metabolite participates in is unclear. *p < 0.05; **p < 0.01

**Fig. 3:**
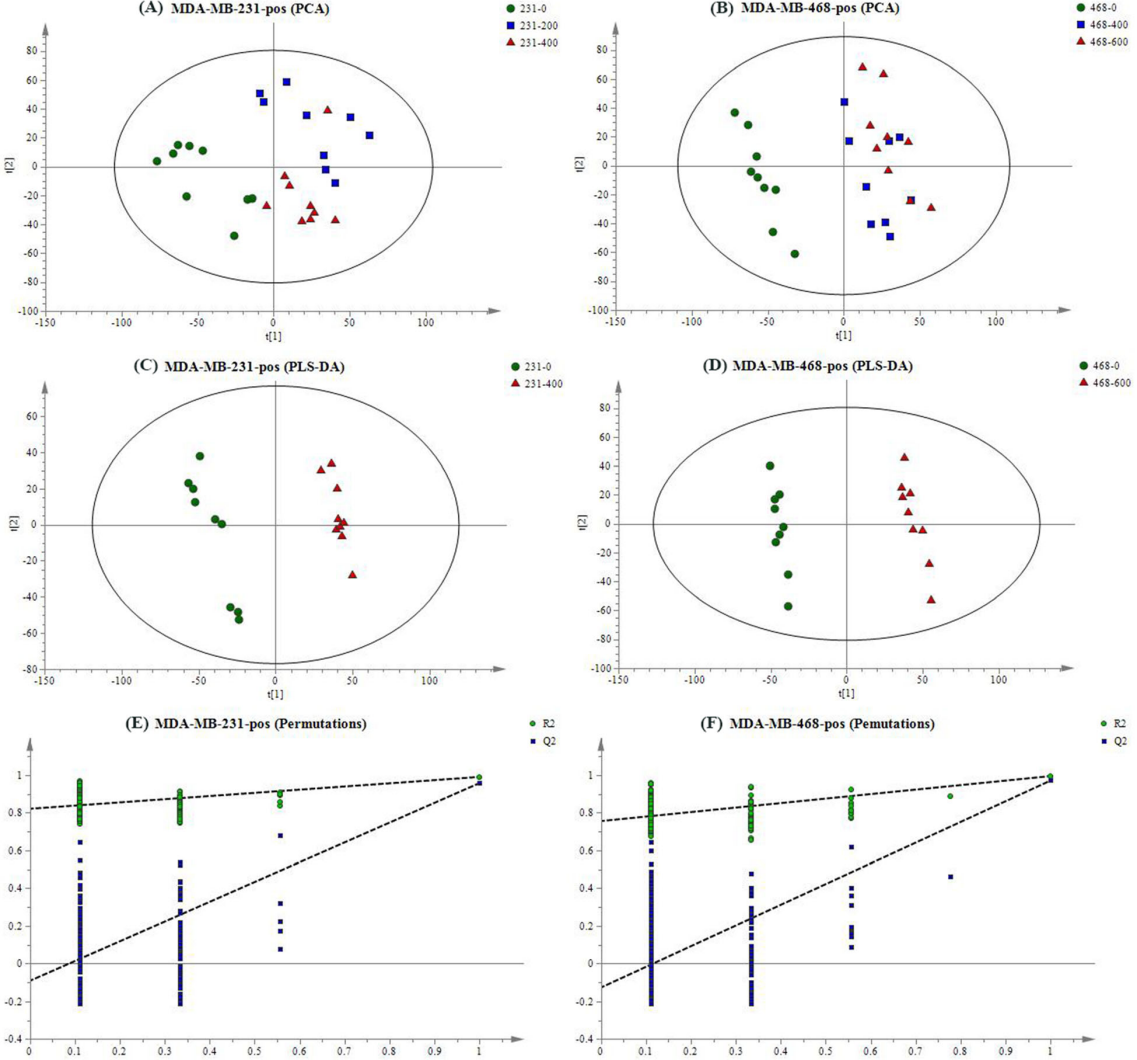
The result of multivariate statistical analysis in positive mode. PCA score plot of MDA-MB-231 cells and MDA-MB-468 cells were showed in **a** and **b**. The green circle represents the control group respectively named 231–0 or 468–0; the blue box represents the middle dose group such as 231–200 or 468–400; the red triangle means the high dose group name 231–400 or 468–600. The EECR treatment groups were obviously distinct with the control groups through the PCA analysis (**a**, **b**). The PLS-DA was performed between the high dose group and the control group resulted in R2X = 0.389, R2Y = 0.991, Q2 = 0.959 in MDA-MB-231 cells (**c**), and R2X = 0.443, R2Y = 0.996, Q2 = 0.972 in MDA-MB-468 cells (**d**). The permutation plot tests the Statistical validation of the PLS-DA model (*n* = 200) showing the values of R^2^ (green circle) and Q^2^ (blue box) (**e**, **f**)

To visualize the overall changes of TNBC cells after the intervention of EECR, PCA was applied in the groups of MDA-MB-231 cell line (named: 231–0, 231–200, 231–400) and MDA-MB-468 cell line (named: 468–0, 468–400, 468–600) separately. Overall, the EECR treatment groups could be obviously differentiated from the control group, both in electron spray ionization positive (ESI^+^) mode (Fig. 3a, b) and negative (ESI^−^) mode (FigS1 A, B), which expressed that the intervention of EECR give rise to the changes of metabolites in TNBC cell lines. In combination with the result of the cell viability analysis, the changed metabolites were valuable for exploring the function mechanism of EECR on TNBC cells. In order to obtain these changed metabolites, groups of 231–0 and 231–400 were selected for PLS-DA, and groups of 468–0 and 468–600 did likewise. In ESI^+^ mode, the groups of 231–0 and 231–400 got a clear separation with R2X = 0.389, R2Y = 0.991, and Q2 = 0.959 (Fig. 3c), the groups of 468–0 and 468–600 had a distinct separation with R2X = 0.443, R2Y = 0.996, and Q2 = 0.972 (Fig. 3d); while in ESI^−^ mode, the PLS-DA score scatter plot showed an obvious discrimination between the group of 231–400 and 231–0 with R2X = 0.421, R2Y = 0.994, and Q2 = 0.942 (FigS1 C), so it is between the group of 468–0 and 468–600 with R2X = 0.432, R2Y = 0.995, and Q2 = 0.962 (FigS1 D). The permutation plot was used to evaluate the PLS-DA model (*n* = 200) (Fig. 3e, f and FigS1 E, F), and showed no-overfitting in these models.

### The identification of significantly differential metabolites

Following the rules of VIP > 1, *p* < 0.05, and FC > 1.2(or FC < 0.8), a total of forty-nine features were identified as significantly differential metabolites. The detailed information of each metabolite was listed in Table 1. Among them, fifteen metabolites were found only via MDA-MB-231 cell line, eleven were detected by means of MDA-MB-468 cell line, and twenty-three were identified in both cell lines.

**Fig. 4:**
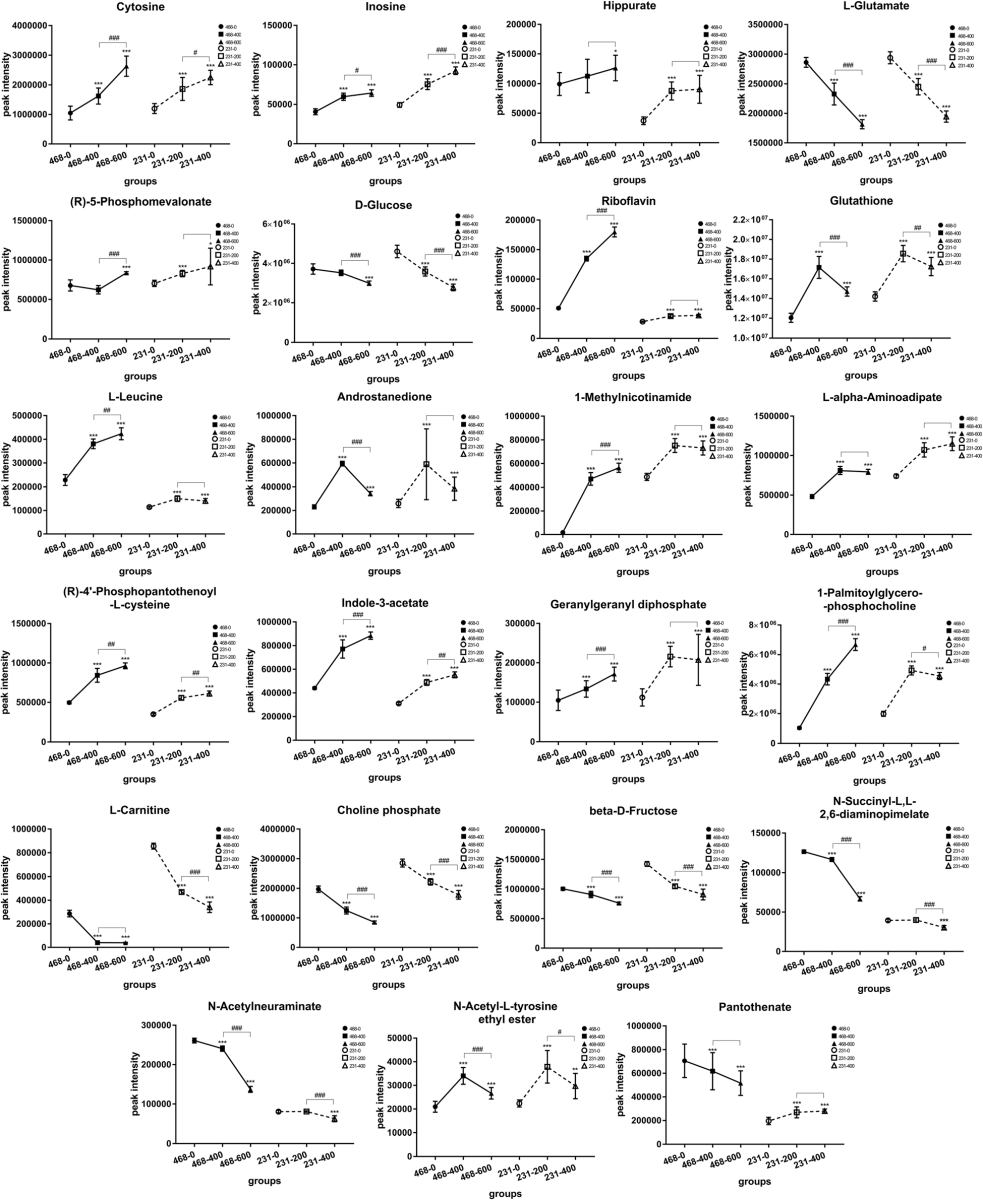
Change trends of twenty-three significantly differential metabolites identified in both cell lines between control groups, middle dose groups, and high dose groups. The solid line represents the result of MDA-MB-468 cell line; the dotted line is on behalf of the result of MDA-MB-231 cell line. The symbol of “*” indicates that the EECR treatment groups are compared statistically with the control groups. The symbol of “^#^” means that the high dose groups are compared statistically with the middle dose groups. The result were given as mean ± SD, *n* = 9, *p < 0.05, **p < 0.01, ***p < 0.001; ^#^*p* < 0.05, ^##^*p* < 0.01, ^###^*p* < 0.001

Except for Pantothenate, the other twenty-two metabolites have the same expression trend in content with statistically significant Fig. 4. Among those, the metabolites being significant up-regulation in both two cell lines included Cytosine, Inosine, Hippurate, (R)-5-Phosphomevalonate, Glutathione, L-Leucine, N-Acetyl-L-tyrosine ethyl ester, Riboflavin, Androstanedione, 1-Methylnicotinamide, L-alpha-Aminoadipate, (R)-4′-Phosphopantothenoyl-L-cysteine, Indole-3-acetate, Geranylgeranyl diphosphate, and 1-Palmitoylglycerophosphocholine. The metabolites being significant down-regulation in both two cell lines contained D-Glucose, L-Glutamate, L-Carnitine, Choline phosphate, beta-D-Fructose, N-Succinyl-L,L-2,6-diaminopimelate, and N-Acetylneuraminate.

### The function mechanism analysis of EECR on TNBC cells

**Fig. 5:**
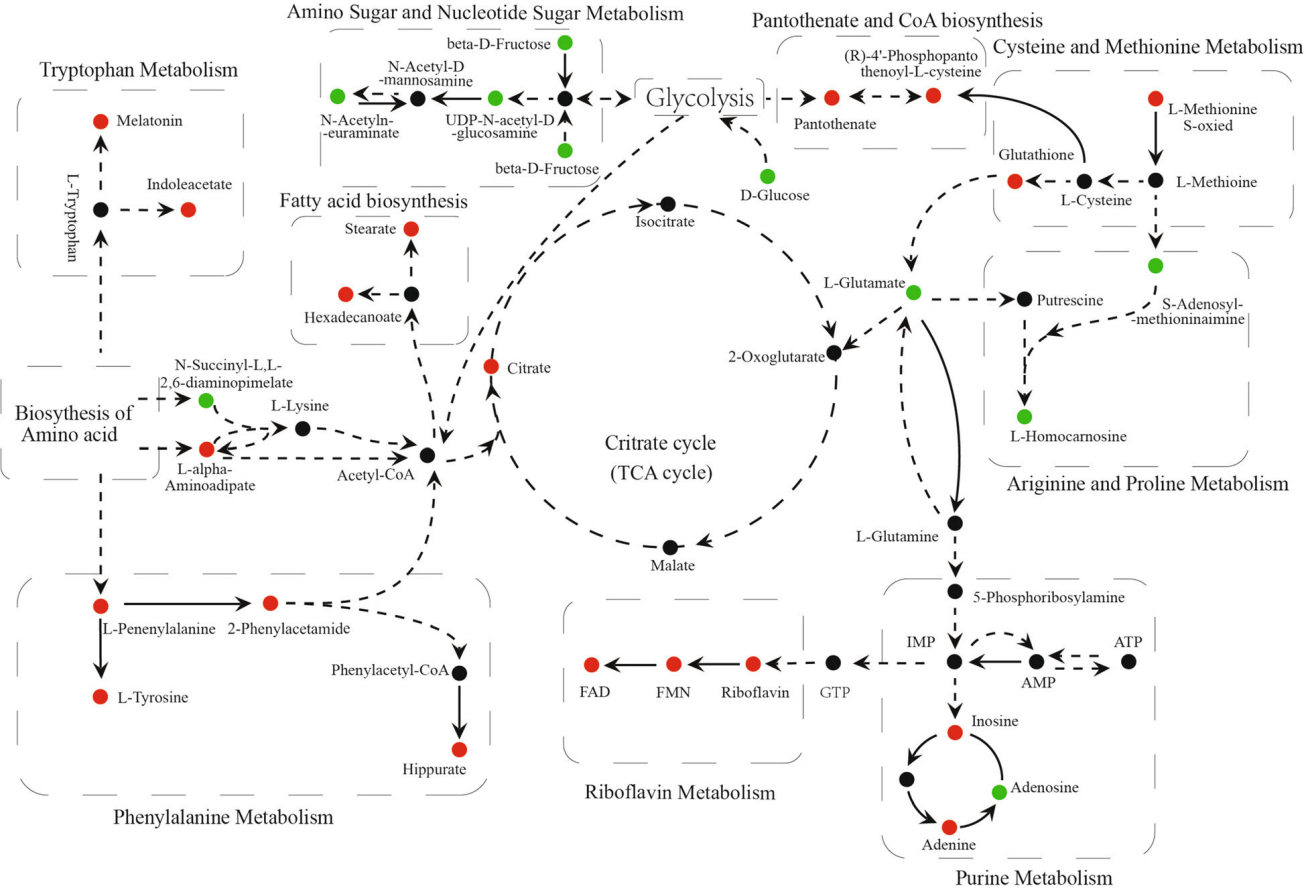
Schematic of metabolic pathway being mapped by significantly differential metabolites. The black line indicates direct reactions between each other, and the dotted line represents more than one reaction between them. The red circle means significantly increased level (*p* < 0.05), while the green circle means the obviously decreased level (*p* < 0.05)

All significantly differential metabolites were searched against the KEGG database (https://www.kegg.jp/) for matching the metabolism pathway. The function mechanism of EECR on TNBC is mainly related to Amino acid metabolism, Riboflavin metabolism, Central carbon metabolism, Purine metabolism, and Amino Sugar and Nucleotide Sugar metabolism. The detailed changes in the main metabolism pathways were visualized in Fig. 5. As shown, metabolites taking part in Amino Sugar and Nucleotide Sugar metabolism and Arginine and Proline metabolism are reduced, while others are generally increased.

## Discussion

The cytotoxicity of EECR on TNBC cells has been reported and is confirmed once again [7, 10]. This research employed cell metabolism to reveal the function mechanism of EECR on TNBC cells at the level of small molecular metabolites and identified forty-nine significant differential metabolites. Both in MDA-MB 231 cell line and MDA-MB 468 cell line, the level of most metabolites mentioned above showed the same variation trend between control group, middle dose group, and high dose group. As shown in Fig. 4, it explained the reason why the EECR induced apoptosis on TNBC cells with dose-dependent manner and confirmed the value of these metabolites in the process of EECR inducing TNBC apoptosis.

By searching for KEGG database, these significant differential metabolites were mapped to such metabolic pathways including Central Carbon metabolism in cancer, Glycolysis, Amino Sugar and Nucleotide Sugar metabolism, Phenylalanine metabolism, Arginine and Proline metabolism, Cysteine and Methionine metabolism, Tryptophan metabolism, Lysine degradation, Purine metabolism, Riboflavin metabolism, Pantothenate and CoA biosynthesis, and Fatty Acid biosynthesis. To sum up, EECR induced TNBC cells apoptosis mainly through five major types of metabolism involving Amino Acid metabolism, Carbohydrate metabolism, Nucleotide metabolism, Lipid metabolism, and metabolism of Cofactors and Vitamins.

### Carbohydrate metabolism

The fore-mentioned Carbohydrate metabolic processes, containing Central Carbon metabolism (TCA cycle) in cancer, Glycolysis, and Amino Sugar and Nucleotide Sugar metabolism, could provide energy for cell proliferation. The high level of citrate and the low level of glucose and L-glutamate were found in EECR treatment groups. Metabolites taking part in Amino Sugar and Nucleotide Sugar metabolism were up-regulated after EECR treatment.

A fundamental difference in the central metabolism exists between cancer cells with normal cells. In 1956, the German Nobel Prize winner Otto Warburg established that cancer cells consumed an abundance of glucose and maintained a high rate of glycolysis even under sufficient oxygen concentrations to support mitochondrial oxidative phosphorylation which was known as “aerobic glycolysis” [19–21]. Citrate, the first production of the TCA cycle, plays a negative feedback regulating role in glycolysis and TCA cycle itself. Citrate slows down or arrests these pathways, but it stimulates the ATP-consuming pathways. The lower level of glucose in EECR treatment groups exactly confirms that breast cancer cells have consumed too much glucose for producing energy compared to the control group. While the citrate at a high level slowed down the Glycolysis in negative feedback manner.

The citrate is not only a key regulator of energy production but an essential metabolic intermediary. It could activate acetyl-CoA carboxylase, the first enzyme for fatty acid synthesis, and provide abundantly Acetyl-CoA for the Fatty Acid biosynthesis [22]. The elevated levels of Stearate and Hexadecanoate participating in the Fatty Acid biosynthesis and benefiting from the activation of Citrate were detected in this study. As a consequence, the ATP consumption was increased.

The EECR acted on Carbohydrate metabolism, breaking the balance between ATP-production and ATP-consumption, resulting in severe energy depletion inside cells, leading to cell growth arrest and cell death.

### Riboflavin metabolism

Riboflavin, as a biological precursor of FAD and FMN which serves as electron carriers for a range of redox reactions, participates in oxidation-reduction reactions in numerous metabolic pathways and in energy production via aerobic respiration [23–25]. Lack of riboflavin has been reported to be associated with an increased risk for malignant tumor [26]. The supplement of Riboflavin enhanced the aerobic respiration in mitochondria which might compete for more substrates and restrain the aerobic glycolysis to some extent [27–29]. After EECR treatment, the levels of Riboflavin and its active coenzymic molecules such as FAD and FMN were all significantly elevated. We suppose that EECR suppresses the aerobic glycolysis by inducing the Riboflavin metabolism to arrest cell proliferation.

### Amino acid metabolism

All amino acids are valuable energy sources or precursors for the manufacture of other important substances. In this study, Amino acid metabolisms containing Phenylalanine metabolism, Cysteine and Methionine metabolism, Tryptophan metabolism, Lysine degradation, and Arginine and Proline metabolism were influenced by EECR. The levels of all significantly changed metabolites, which take part in Phenylalanine metabolism, Cysteine and Methionine metabolism, Tryptophan metabolism, or Lysine degradation, were up-regulated, whereas those participating in Arginine and Proline metabolism were down-regulated.

Abnormal Tryptophan metabolism is reported in a variety of cancers [30–33]. Serotonin and melatonin are two vital products of Tryptophan metabolism. The low-expression of indoleacetate, a breakdown product of tryptophan metabolism, was also reported in breast cancer [31]. In this study, the treatment of EECR reversed the trend of indoleacetate which show a higher level in EECR treatment group. The mechanism of serotonin was extremely complex in breast cancers, which seems to transfer its tumor-suppressive actions into a tumor-promoting effect, accompanied by a significant increase in Serotonin synthesis [34, 35]. The therapies on the serotonin system will provide valuable therapeutic method for breast cancer. Melatonin is a downstream product of serotonin. And the reduction of melatonin has been reported in patients with malignant tumors [32]. Gamal H. et al. reported that melatonin prevented breast cancer metastasis through inhibiting DJ-1/KLF17/ID-1 signaling pathway [36]. In this study, the level of melatonin was enhanced after the treatment of EECR. The significant increase of melatonin reversed the expression trend in malignant tumors. Although the specific mechanism is still unclear, we predict that the EECR acts on the serotonin system, in which it accelerated the transformation of serotonin to melatonin.

Phenylalanine and tyrosine both are the precursors of the catecholamines which is a kind of neurotransmitters acting as adrenalin-like substances. The alterations of genes related to Phenylalanine metabolism were reported in breast cancer tissue [37]. In this research, all significantly differential metabolites matched to the Phenylalanine metabolism were found at a higher level after the EECR treatment. Some reports suggested that a high concentration of phenylalanine and its metabolites cloud induce cell apoptosis [38, 39]. We suppose the apoptosis is related to the higher level of Phenylalanine and its downstream metabolites.

In addition, breast cancer cells were reported as methionine-dependent cell line, which means the low expression of methionine limited their proliferation [40]. S-adenosylhomocysteine and methionine were two critical products between the methionine cycle, the expressions of S-adenosylhomocysteine and methionine depend on each other. The low level of S-adenosylhomocysteine was found in this study reflecting a low concentration of methionine which indicated the limited proliferation of TNBC cells.

Glutathione, synthesized from three amino acids: glycine, glutamate, and cysteine, abundantly and widely distributes throughout different cell types. The high level of glutathione has a complicated role in both cancer and antineoplastic therapy which plays a vital role in the elimination of carcinogens while increases the resistance to cancer chemotherapeutic [41]. The remarkably lower level of Glutamate being a substance of glutathione was detected in this study. Consequently, the synthesis of glutathione could be feedback-inhibited by the short of Glutamate. Although further research is essential, we establish that EECR could decrease the level of Glutathione through glutathione cycle at last.

Glutamine, a product of glutamate, is a significant energy source in general and particularly for malignant cells and plays a vital role as a source of nitrogen for the synthesis of nucleotides [42]. In this research, the lower level of glutamine was predicted by lower level of glutamate. The Purine metabolites, a kind of Nucleotide metabolism, provide the necessary energy and cofactors to promote cell survival and proliferation. Owing to their glutamine addiction, depletion of glutamine may arrest the proliferation and induce the apoptosis of TNBC cells [43].

In addition to considering the specific function of each amino and its metabolites, the action of ATP-consumption of each amino acid metabolism was taken into account for the depletion of energy, resulted in TNBC cell apoptosis ultimately.

## Conclusion

In this study, we applied cell metabolism to detect the function mechanism of EECR on TNBC and found forty-nine significant differential metabolites which mapped to Central Carbon metabolism in cancer, Glycolysis, Amino Sugar and Nucleotide Sugar metabolism, Phenylalanine metabolism, Arginine and Proline metabolism, Cysteine and Methionine metabolism, Tryptophan metabolism, Lysine degradation, Purine metabolism, Riboflavin metabolism, Pantothenate and CoA biosynthesis, and Fatty Acid biosynthesis.

Among these significant differential metabolites, twenty-three were found in both two TNBC cell lines, and twenty two metabolites expressed the same change trend in content between control group, middle dose group, and high dose group, which explained the reason why the EECR induced apoptosis on TNBC cells with dose-dependent manner and confirmed the value of these metabolites in the process of EECR inducing TNBC apoptosis.

Through a systematic metabolism analysis, EECR impacted the energy metabolism of TNBC cells by arresting the pathways of Carbohydrate metabolism such as Central carbon metabolism in cancer, aerobic glycolysis, and Amino sugar and nucleotide sugar metabolism, whereas accelerating the pathways of ATP-consumption including Amino acids metabolism, Fatty acid metabolism, Riboflavin metabolism and Purine metabolism which broke the balance between ATP-production and ATP-consumption, resulted in severe ATP depletion inside cells, arrested the proliferation and induced the apoptosis of TNBC cells.

In conclusion, EECR induces apoptosis mainly by affecting the energy metabolism. This research reveals that EECR has great potential in the clinical treatment of TNBC with fewer toxic and side effects which need further study.

## Supplementary information

**Additional file 1: Figure S1.** The result of multivariate statistical analysis in negative mode. PCA score plot of MDA-MB-231 cells and MDA-MB-468 cell were showed in A and B. The green circle represents the control group respectively named 231–0 or 468–0; the blue box represents the middle dose group such as 231–200 or 468–400; the red triangle means the high dose group name 231–400 or 468–600. The EECR treatment groups were obviously distinct with the control groups through the PCA analysis (A, B). The PLS-DA was performed between the high dose group and the control group resulted in R2X = 0.421, R2Y = 0.994, and Q2 = 0.942 in MDA-MB-231 cells (C), and R2X = 0.432, R2Y = 0.995, and Q2 = 0.962 in MDA-MB-468 cells (D). The permutation plot tests the Statistical validation of the PLS-DA model (*n* = 200) showing the values of R2 (green circle) and Q2 (blue box) (E, F).

## Data Availability

The datasets used and/or analysed during the current study available from the corresponding author on reasonable request.

## Abbreviations

TNBC: Triple-negative breast cancer
EECR: The ethanol extract of *Cyperus rotundus* L
UPLC-Q-TOF-MS/MS: Ultra-high performance liquid chromatography coupled with quadrupole-time-of-flight mass spectrometry
ER: Estrogen receptor
PR: Progesterone receptor
Her-2: Human epidermal growth factor receptor-2
DMEM: Dulbecco’s modified Eagle’s medium
FBS: Fetal bovine serum
PBS: Phosphate-buffered saline
DMSO: Dimethyl sulfoxide
CCK-8: Cell Counting Kit-8 assay
OD: Optical density
PI: Propidium Iodide
QC: Quality control
PCA: Principal component analysis
PLS-DA: Partial least squares discriminant analysis
FC: Fold Change
RSD: The relative standard deviation
BPCs: Base peak chromatograms
FAD: Flavin adenine dinucleotide
FMN: Riboflavin-5-phosphate
