# Supplementary figures and images for: Cell metabolomics to study the function mechanism of Cyperus rotundus L. on triple-negative breast cancer cells
Source: BMC Complement Med Ther. 2020 Aug 26;20:262. doi: 10.1186/s12906-020-02981-w (PMC7449030; doi:10.1186/s12906-020-02981-w)

**
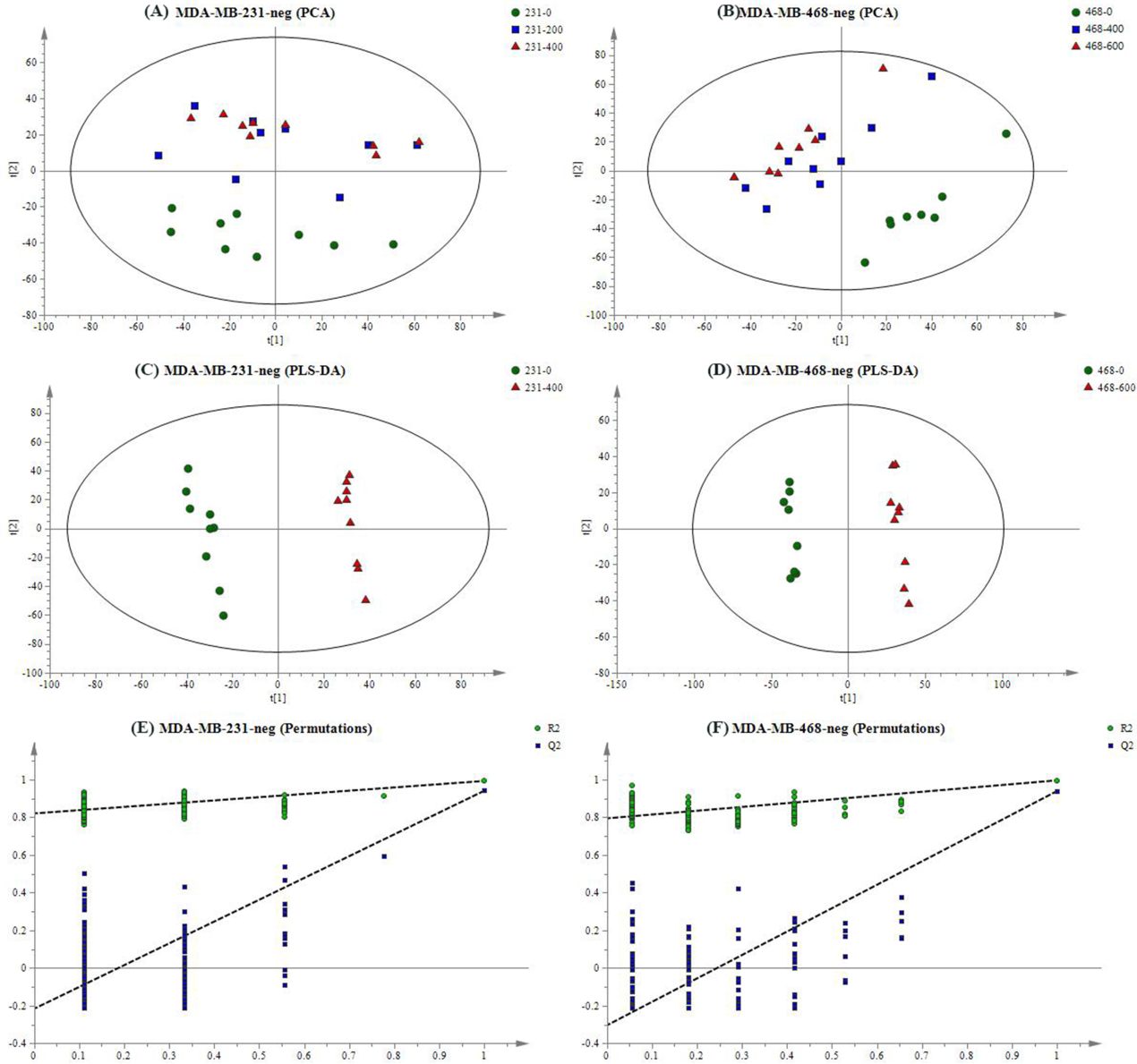
**

Supplement: Supplementary file 1 — Additional file 1: Figure S1. The result of multivariate statistical analysis in negative mode. PCA score plot of MDA-MB-231 cells and MDA-MB-468 cell were showed in A and B. The green circle represents the control group respectively named 231–0 or 468–0; the blue box represents the middle dose group such as 231–200 or 468–400; the red triangle means the high dose group name 231–400 or 468–600. The EECR treatment groups were obviously distinct with the control groups through the PCA analysis (A, B). The PLS-DA was performed between the high dose group and the control group resulted in R2X = 0.421, R2Y = 0.994, and Q2 = 0.942 in MDA-MB-231 cells (C), and R2X = 0.432, R2Y = 0.995, and Q2 = 0.962 in MDA-MB-468 cells (D). The permutation plot tests the Statistical validation of the PLS-DA model (n = 200) showing the values of R2 (green circle) and Q2 (blue box) (E, F). [file 12906_2020_2981_MOESM1_ESM.docx]
